# Supplementary figures and images for: Proteomic analysis reveals microvesicles containing NAMPT as mediators of radioresistance in glioma
Source: Life Sci Alliance. 2023 Apr 10;6(6):e202201680. doi: 10.26508/lsa.202201680 (PMC10087103; doi:10.26508/lsa.202201680)

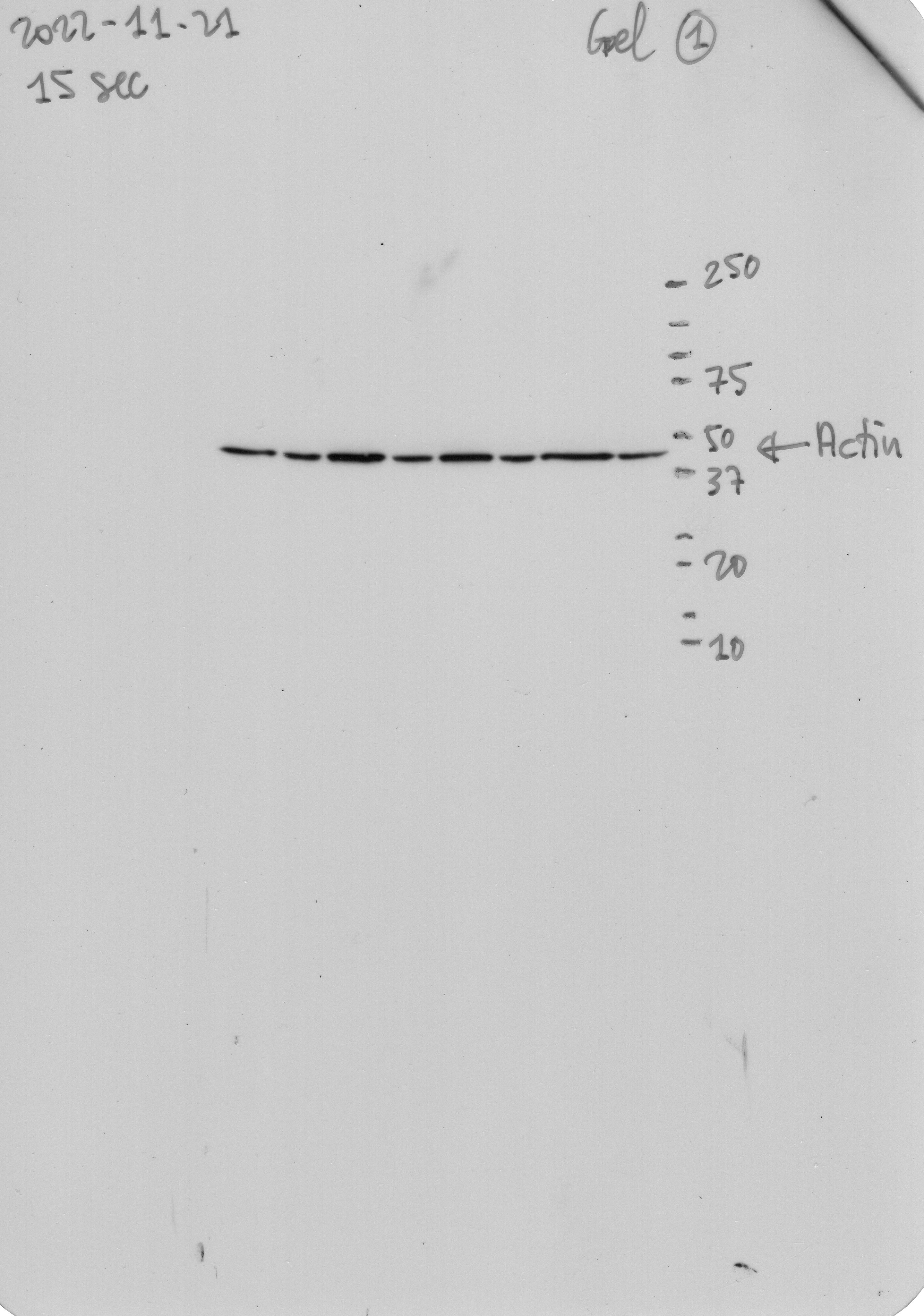

Supplement: Supplementary file 4 [file LSA-2022-01680_SdataF2.zip › SourceDataForFigure2/figure_2I_WB/actin.jpg]

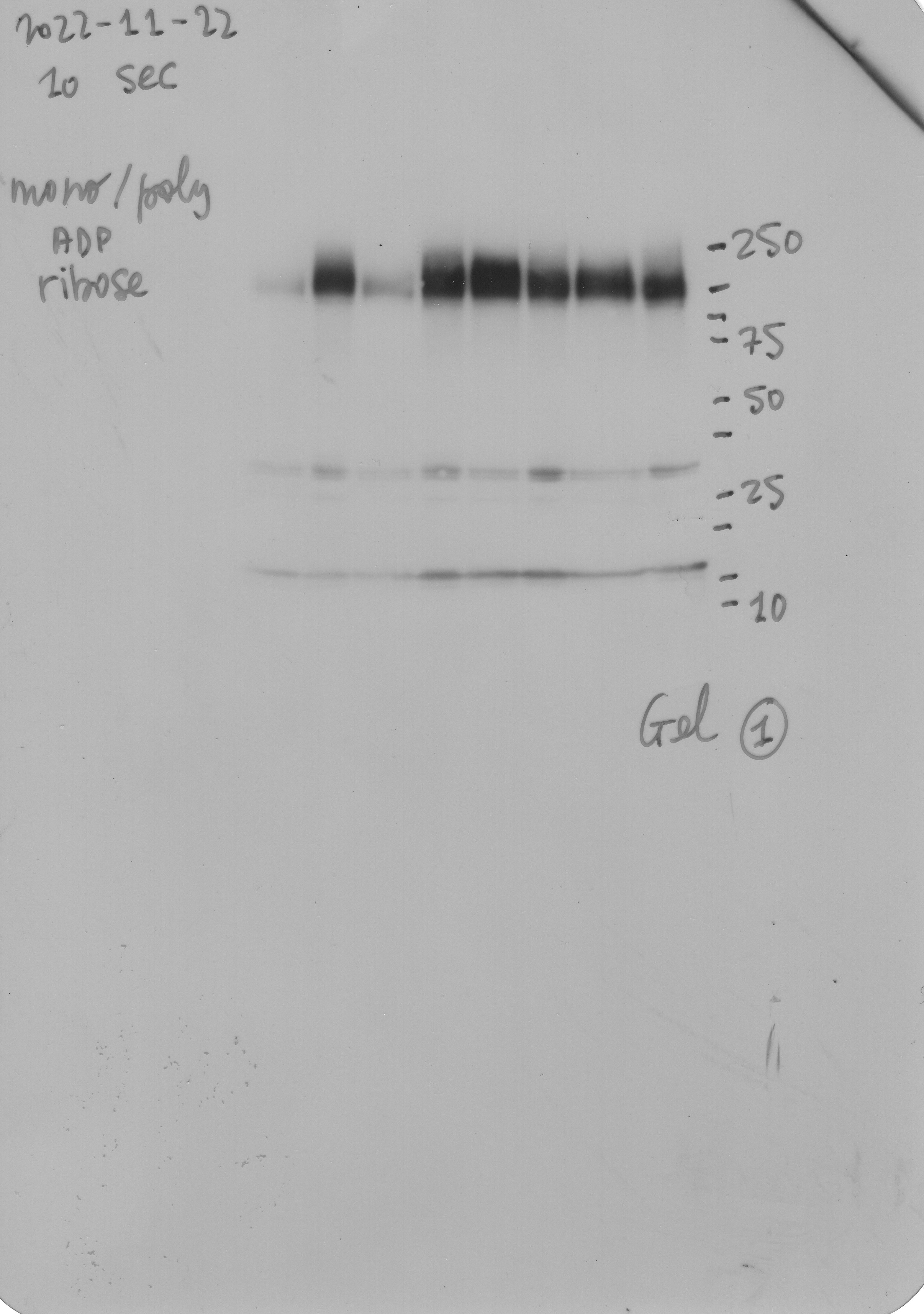

Supplement: Supplementary file 4 [file LSA-2022-01680_SdataF2.zip › SourceDataForFigure2/figure_2I_WB/poly-mono_ADP_ribose.jpg]

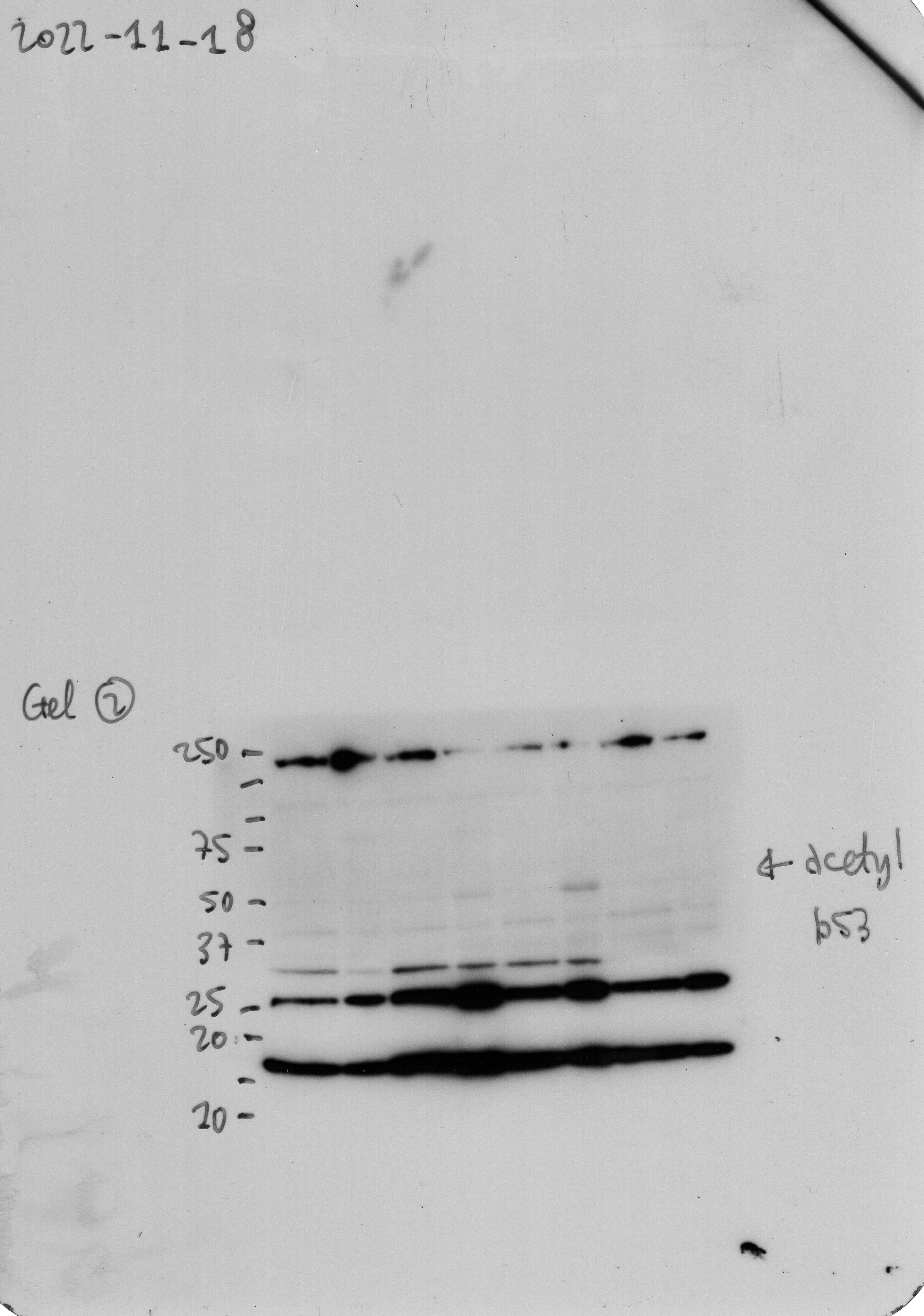

Supplement: Supplementary file 4 [file LSA-2022-01680_SdataF2.zip › SourceDataForFigure2/figure_2I_WB/acetyl-p53.jpg]

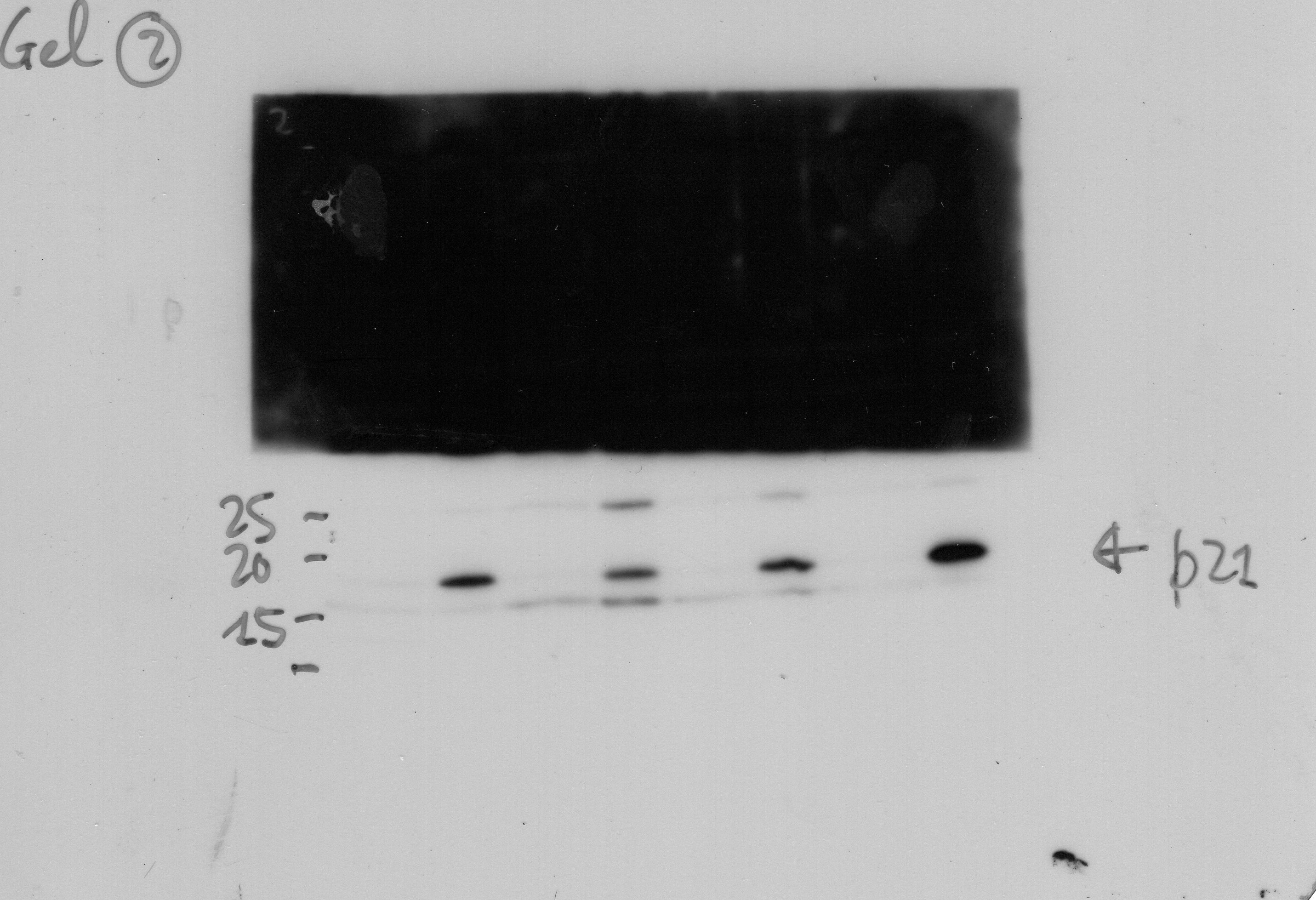

Supplement: Supplementary file 4 [file LSA-2022-01680_SdataF2.zip › SourceDataForFigure2/figure_2I_WB/p21.jpg]

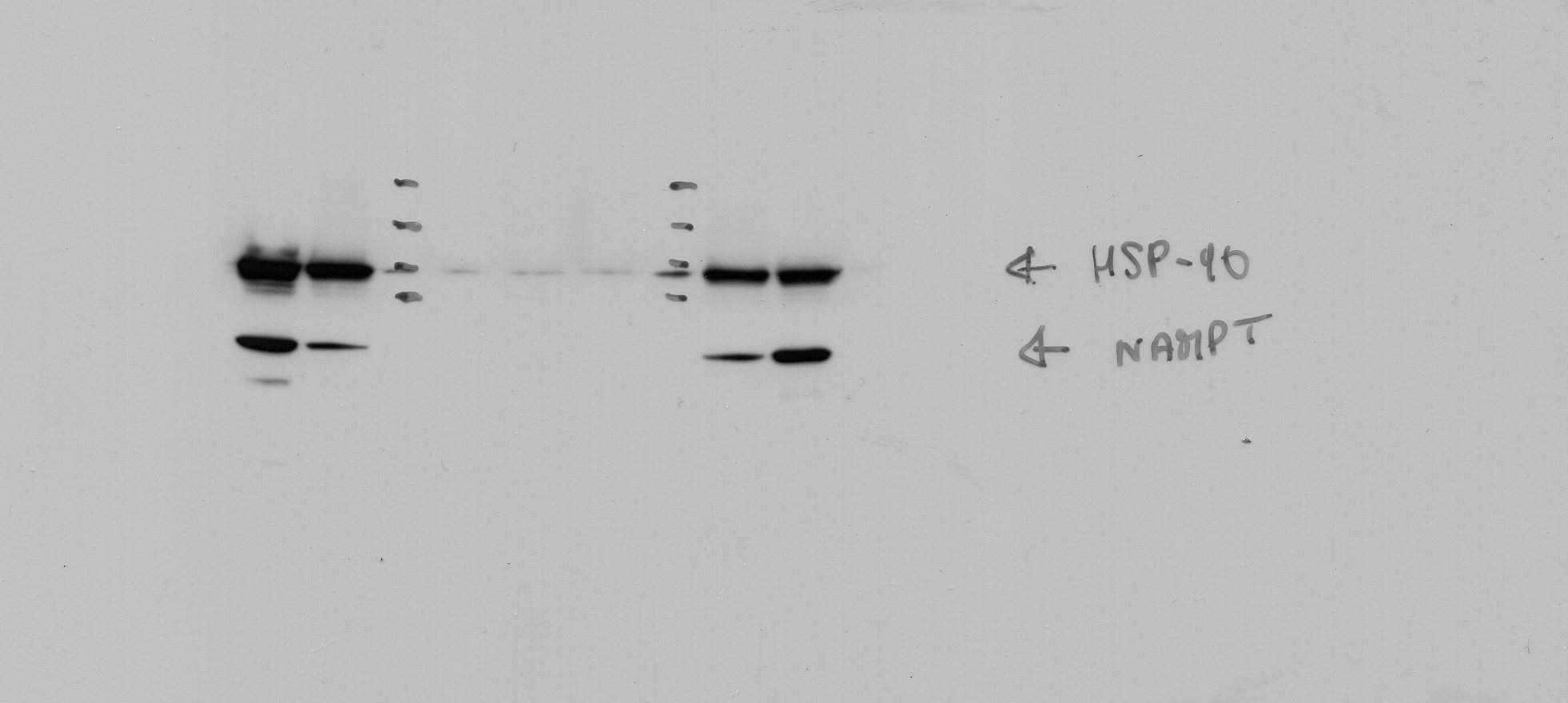

Supplement: Supplementary file 7 [file LSA-2022-01680_SdataF3.zip › SourceDataForFigure3/figure_3F_WB/HSP90_short_exposure.jpg]

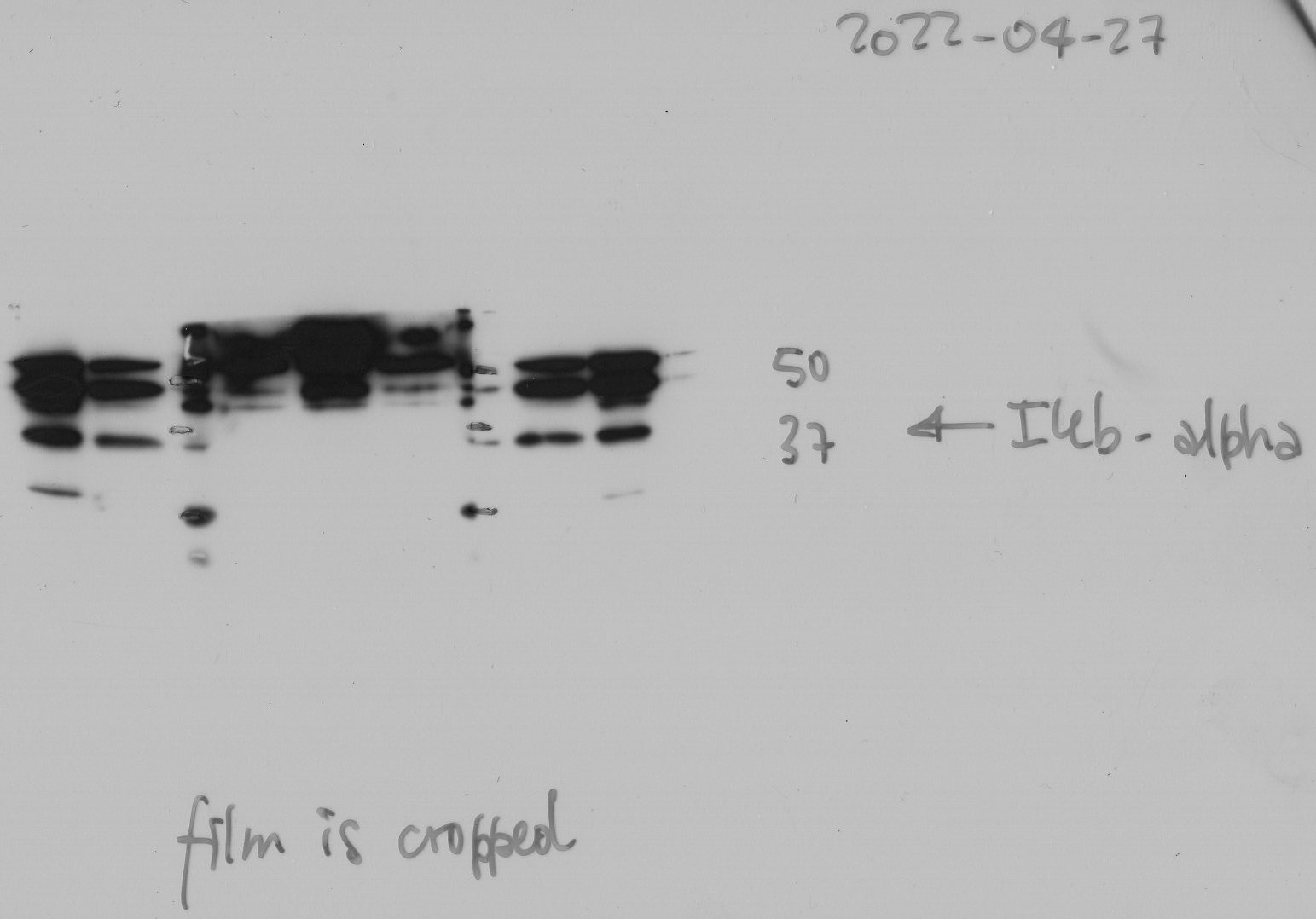

Supplement: Supplementary file 7 [file LSA-2022-01680_SdataF3.zip › SourceDataForFigure3/figure_3F_WB/IKB-alpha.jpg]

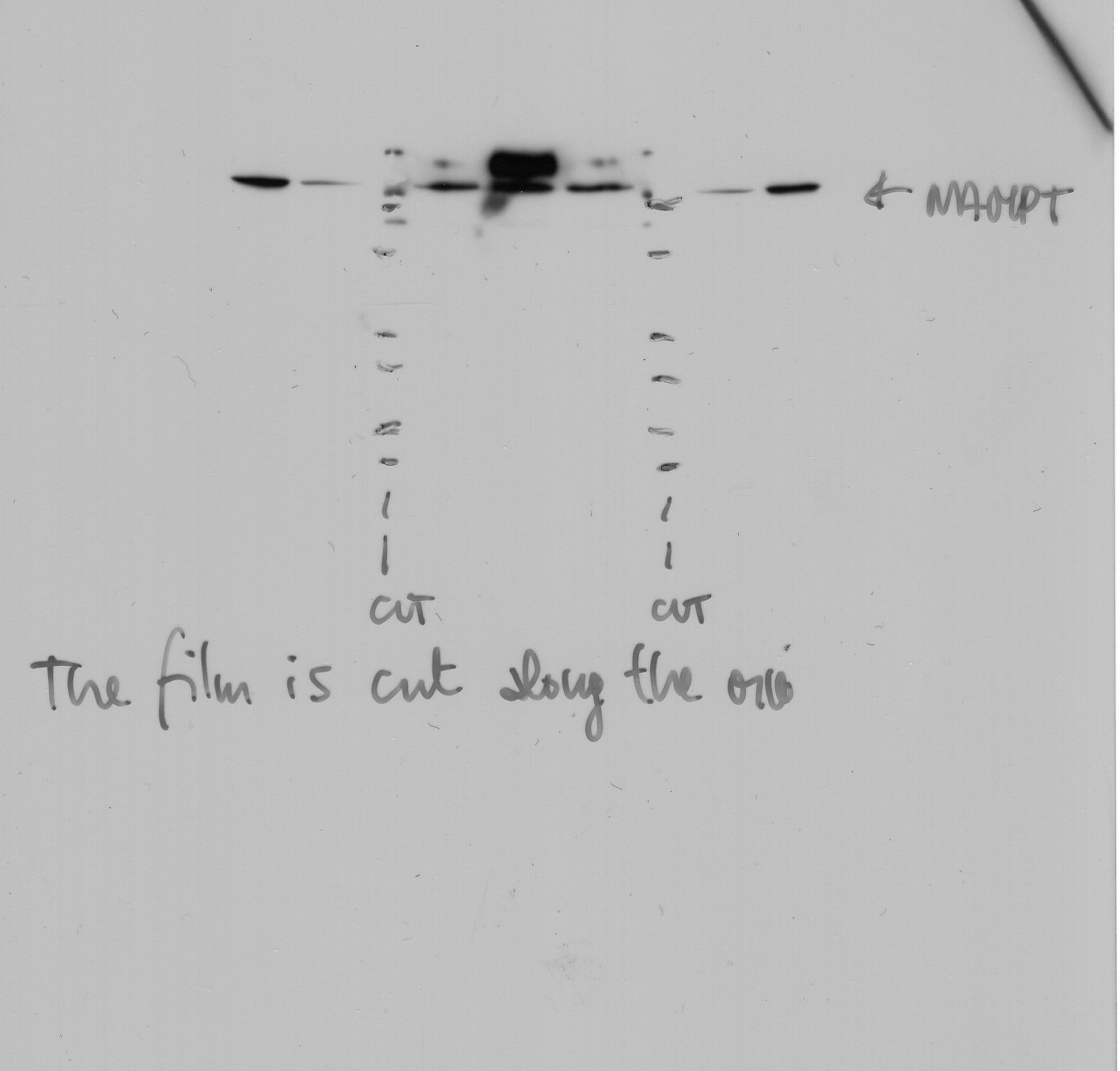

Supplement: Supplementary file 7 [file LSA-2022-01680_SdataF3.zip › SourceDataForFigure3/figure_3F_WB/NAMPT.jpg]

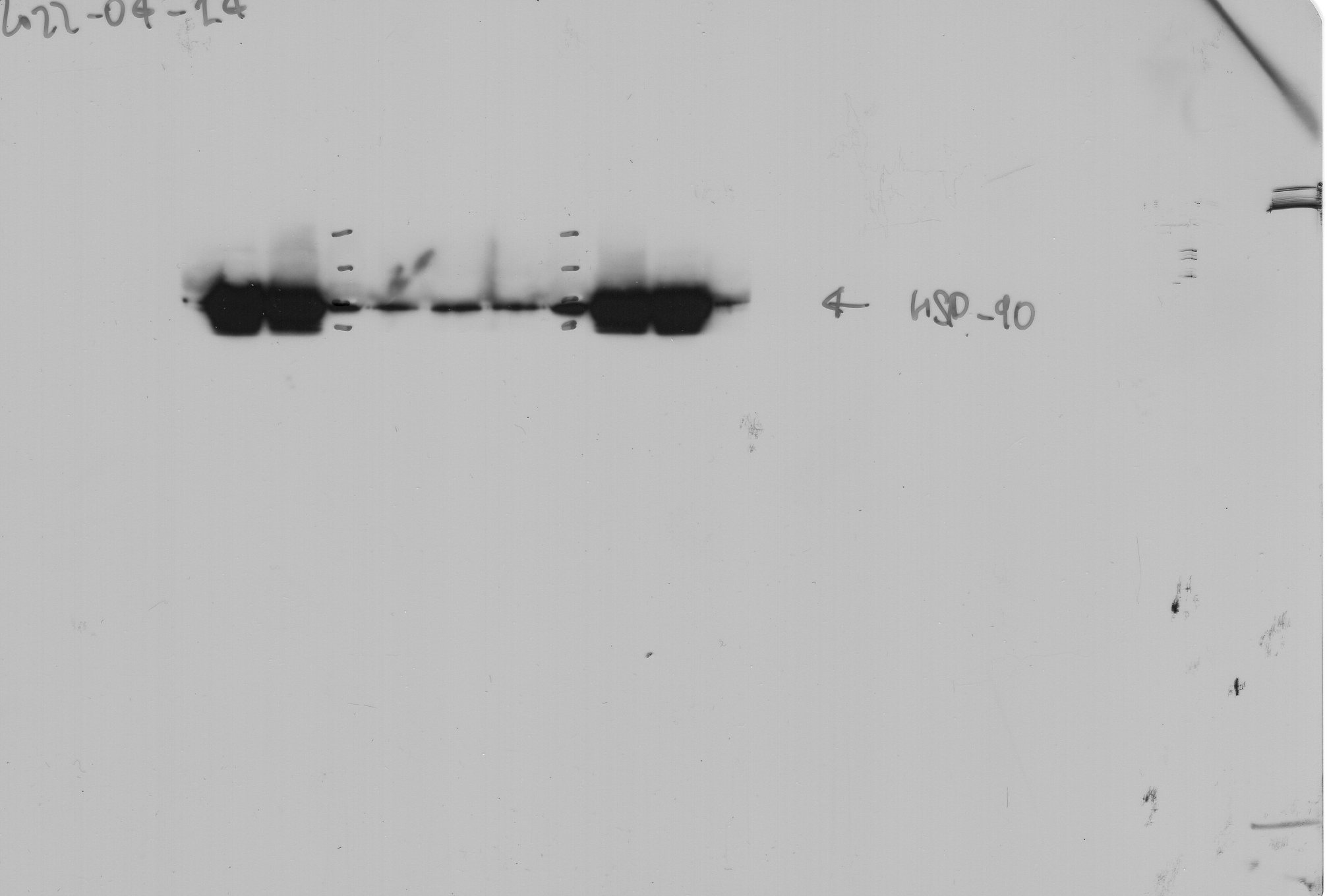

Supplement: Supplementary file 7 [file LSA-2022-01680_SdataF3.zip › SourceDataForFigure3/figure_3F_WB/HSP90_long_exposure.jpg]

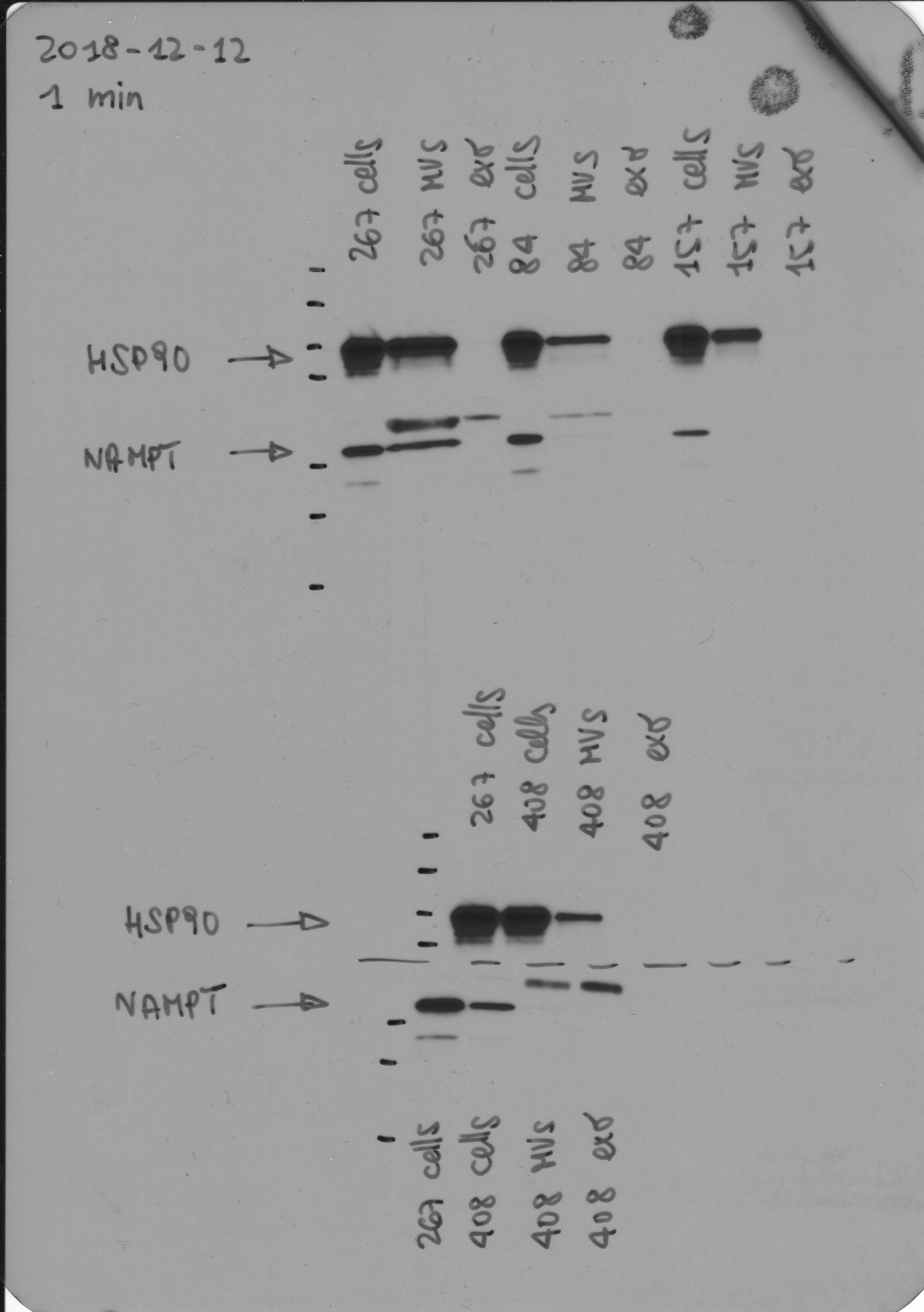

Supplement: Supplementary file 7 [file LSA-2022-01680_SdataF3.zip › SourceDataForFigure3/figure_3D_WB/HSP90-NAMPT 1 min.tif]

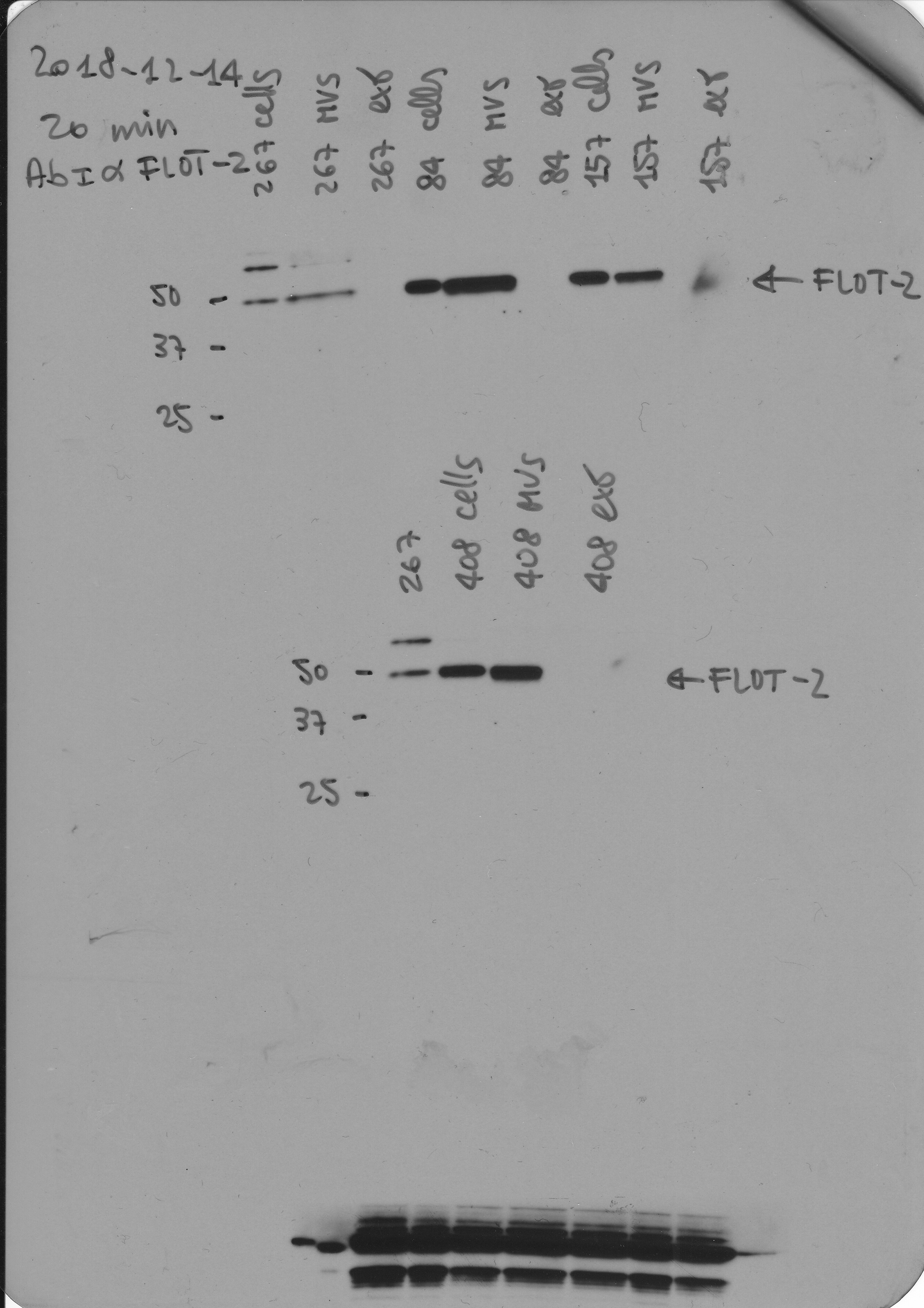

Supplement: Supplementary file 7 [file LSA-2022-01680_SdataF3.zip › SourceDataForFigure3/figure_3D_WB/FLOT-2.tif]

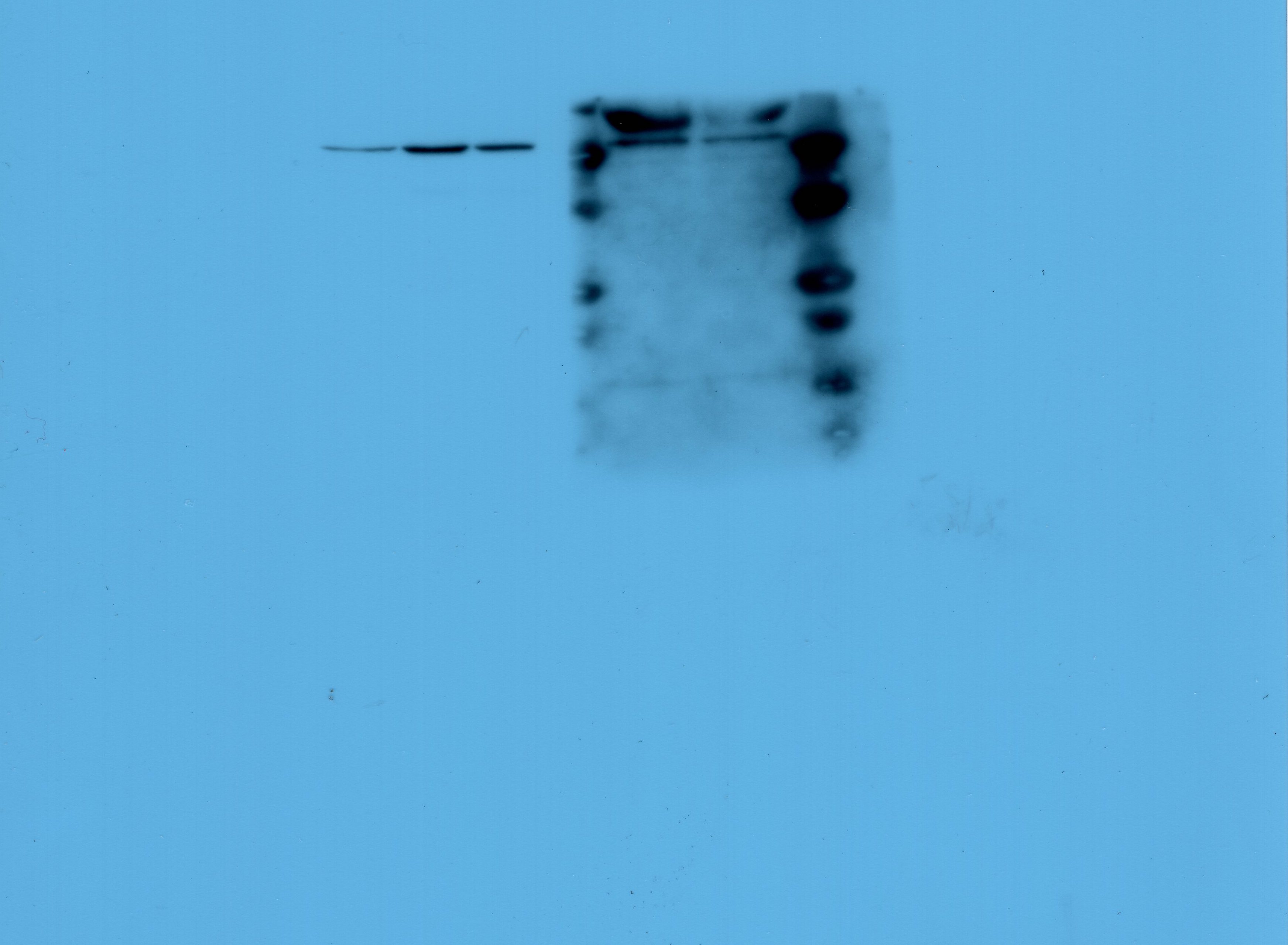

Supplement: Supplementary file 8 [file LSA-2022-01680_SdataF4.zip › SourceDataForFigure4/Figure_4C/NAMPT_in_MVs.jpg]

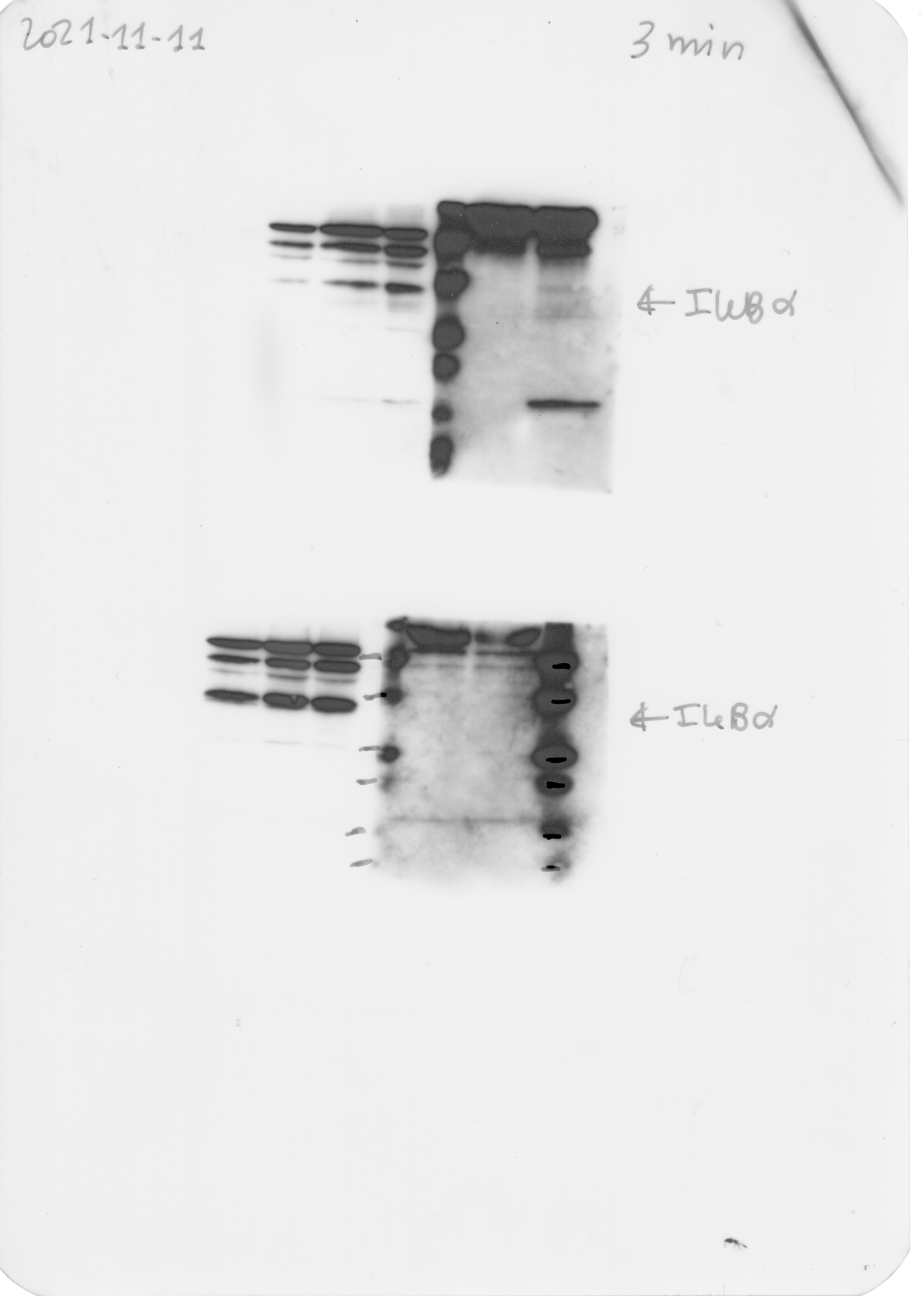

Supplement: Supplementary file 8 [file LSA-2022-01680_SdataF4.zip › SourceDataForFigure4/Figure_4C/IKBalpha.jpg]

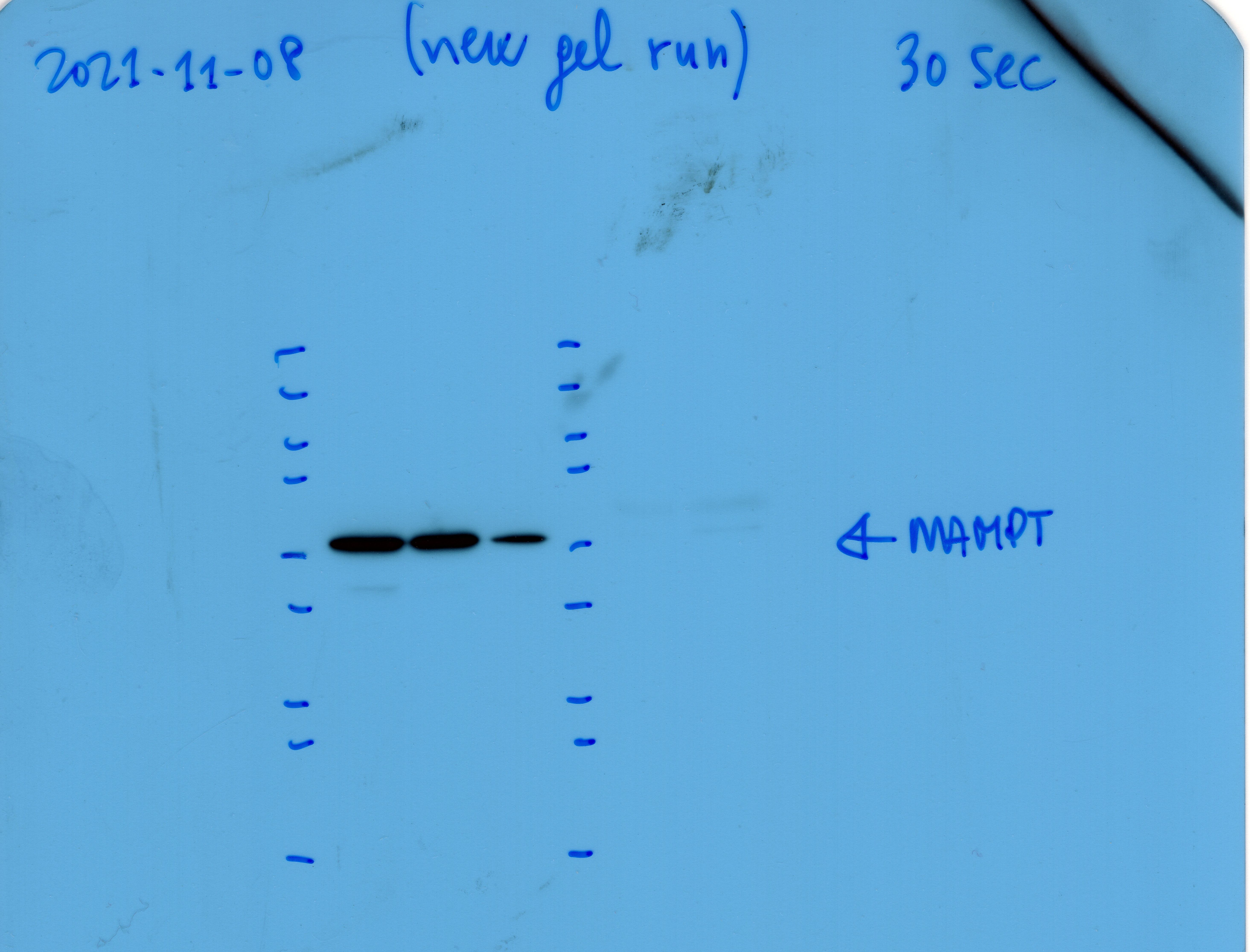

Supplement: Supplementary file 8 [file LSA-2022-01680_SdataF4.zip › SourceDataForFigure4/Figure_4C/NAMPT_in_WC.jpg]
